# Mental Disease after an Operation upon a Fistula

**Published:** 1885-12

**Authors:** W. P. Wesselhœft


### MENTAL DISEASE AFTER AN OPERATION UPON A FISTULA.

April 4th was called to see T. P., Boston Highlands, æt. thirty-nine, dark eyes and complexion, considerably emaciated, his face deeply pock-marked in consequence of variola, which he had many years ago. Sitting on the sofa, his head hanging forward, lower jaw dropped, the tongue lying loosely between the teeth, saliva running from the mouth, his eyes without expression, he has the full appearance of an idiot. His articulation is very imperfect, the tongue lolling about in his mouth, with only occasionally an intelligible word. Is uneasy and restless, his eyes rolling vacantly from object to object, frequently endeavoring to rise, which is done with great effort and awkwardness, and after getting on to his feet the *body bends toward the left* to such an extent that his keeper is obliged to support him from falling over toward this side. In walking, drags his feet, and the direction of his steps is always *toward the left.* Is entirely unable to feed himself, dropping his food into his lap and out of his mouth, must be fed, and seems quite indifferent to food.

Upon close questioning, I find his left arm and left leg seem more useless than the right, although this is not very apparent to me. He seems impressed that he is followed by enemies who are trying to harm him, attempts to leave the room as if frightened by visions close behind him.

It was entirely impossible to get an intelligent answer from the patient, but his wife's account is as follows:

Has been "doctoring" for costiveness for more than a year; about three months ago a fistula in ano appeared, which was operated upon eight weeks ago. A few days after the operation complained of his head, particularly pain in *left temple* and occiput, aching pain in lumbar region ; five weeks ago came home from his work feeling dizzy, faint, and nauseated, talked incoherently, and soon afterward used a language no one could understand, as it were a foreign tongue ; has been entirely without mind since. Will frequently cry and whine, then laugh in the silliest manner. Has not slept at all nights, sleeps only for a minute or two at a time during the day. Is often violent at nights, so that he can with difficulty be kept in bed ; endeavors to climb up the bedpost, and grasp at imaginary objects ; is indifferent to food, but will eat a little when fed. After the most powerful drugs he has small costive operations, only once in six or seven days, voided with great difficulty.

This patient was seen and treated by three allopathic physicians, one a physician from an insane asylum. They gave up the case as incurable, and advised the patient to be taken to an asylum. In their opinion he could live but a short time.

The wife, whom I had relieved of sick headache some years ago, sent for me. I gave her no hope, but required a month's time to try a remedy which seemed strongly indicated. Should he grow more troublesome and boisterous, it would then be time to send him to an asylum.

The pathogenesis of *Lachesis* has the most striking similarity with the prominent features of this case, of which I will here mention the *left-sided* affection of head and limbs, the dropping of the lower jaw, the paralytic condition of the tongue, the lolling speech, and, above all, the symptoms of the sensorium.

On the 4th of April I gave a dose of *Lachesis*$^{200th}$ (Dunham), dissolved in six tablespoonfuls of water, a tablespoonful to be taken every four or five hours until used up.

On the 8th of April, four days later, I saw him again ; has slept two hours the second night, speaks better, articulates more distinctly, answers in a vague, dreamy way, is still followed by enemies, and endeavoring to get out of the house, broke a large pane of glass in the front door ; can feed himself better, and walks with less inclination toward the left ; had a good stool the evening before. *Sac. lac.*

April 12th.—Reports a great deal better, speaks connectedly, horrid visions coming up through the floor of the room occasionally, continual pain in left temple, but less severe, has had three good operations. *Sac. lac.*

April 16th.—Talks quite rationally ; says he has lost all his fancies and visions since the last two days; slight pain in left temple; backache nearly gone; two good stools; has been out to walk, and does not require the assistance of his nurse. *Sac. lac.*

April 22d.—Complains of left temple, as if a screw were being driven into it; forgets recent occurrences; dizziness every afternoon; appetite excellent, speaks well. *Lachesis*$^{200th}$, in water, every six hours a tablespoonful, for twenty-four hours. In a week after he rode out, and is now, May 18th, as well as he ever was, and much better than for two years. The only difficulty he complains of is a soreness in the rectum, lasting several hours after an operation. I told him this was the most favorable symptom he could mention, and if he were fortunate enough to have the fistula reappear would never be troubled with his head again, even if it took *years* to cure that affection strictly *homœopathically*.

W. P. Wesselhœft, M. D.

